# Enhancing Artificial Bee Colony Algorithm with Self-Adaptive Searching Strategy and Artificial Immune Network Operators for Global Optimization

**DOI:** 10.1155/2014/438260

**Published:** 2014-02-18

**Authors:** Tinggui Chen, Renbin Xiao

**Affiliations:** ^1^College of Computer Science & Information Engineering, Zhejiang Gongshang University, Zhejiang Province, Hangzhou 310018, China; ^2^Institute of Systems Engineering, Huazhong University of Science and Technology, Hubei Province, Wuhan 430074, China

## Abstract

Artificial bee colony (ABC) algorithm, inspired by the intelligent foraging behavior of honey bees, was proposed by Karaboga. It has been shown to be superior to some conventional intelligent algorithms such as genetic algorithm (GA), artificial colony optimization (ACO), and particle swarm optimization (PSO). However, the ABC still has some limitations. For example, ABC can easily get trapped in the local optimum when handing in functions that have a narrow curving valley, a high eccentric ellipse, or complex multimodal functions. As a result, we proposed an enhanced ABC algorithm called EABC by introducing self-adaptive searching strategy and artificial immune network operators to improve the exploitation and exploration. The simulation results tested on a suite of unimodal or multimodal benchmark functions illustrate that the EABC algorithm outperforms ACO, PSO, and the basic ABC in most of the experiments.

## 1. Introduction

With the rapid development of communication technology, computer technology, and network technology, humans put forward higher demand for efficient intelligent technologies. However, in view of the complexity, constraint, and nonlinearity of practical issues, searching for all kinds of emerging intelligent computing technologies for solving large and complex problems has been paid attention by more and more scholars.

As one of typical intelligent computing approaches, swarm intelligence that combines biology and social based heuristics has become a research interest to many research scientists of related fields in recent years. It is based on the collective behavior of social insects, flock of birds, or schools of fish. The key components of swarm intelligence are self-organization and division of labor. In a self-organization system, each of the covered units may respond to local stimuli individually and act together to accomplish a global task via division of labor without a centralized supervision. The entire system can adapt to internal and external changes efficiently [1, 2]. Particle swarm optimization (PSO) algorithm introduced by Hsieh et al. in 2008 [3] can be thought of as a typical swarm whose individual agents are birds and has been widely used in all kinds of combination optimization problems [4–7]. What is more, other algorithms such as ant colony optimization (ACO) [8, 9] and artificial immune network (aiNet) [10, 11] can also be considered as subfields of swarm intelligence.

Nowadays, an artificial bee colony (ABC) algorithm, inspired by the intelligent foraging behavior of honey bees, was proposed by Karaboga [12]. Due to its simplicity and ease of implementation, the ABC algorithm has captured much attention and has been widely applied to solve many practical optimization problems such as supply chain management [13] and scheduling optimization [14]. In addition, a set of well-known numerical comparisons have demonstrated that the performance of ABC algorithm is competitive to other intelligent ones including genetic algorithm (GA), PSO, differential evolution (DS), and evolution strategy (ES) although it uses fewer control parameters [15–17].

However, similar to other intelligent algorithms, the ABC still has some limitations. For example, the convergence speed of ABC is slow because of its stochastic nature. What is more, ABC can easily get trapped in the local optimum when handing in functions that have a narrow curving valley, a high eccentric ellipse, or complex multimodal functions [18]. All these insufficiencies prevent the further applications of the ABC algorithm.

Therefore, in this work, some modifications to the standard ABC algorithm are introduced for global optimization of numerical functions. Firstly, ABC algorithm is extended by employing the chaotic systems and the diversity-based method when producing the initial population. Next, self-adaptive searching strategy is incorporated into the employed bee search process to improve the exploitation. In addition, the enhanced algorithm retains the main steps of ABC and incorporates an aiNet-based search technique, where aiNet algorithm has powerful multimodal searching ability as well as good stabilization due to its negative selection and network compression operators. Therefore, ABC and aiNet have complementary advantages, and a hybrid of the two may be a possible strategy to improve the performances of ABC.

The remainder of this paper is organized as follows. In Section 2, the works related to the ABC algorithm are summarized. In Section 3, the basic ABC algorithm is described.  Section 4 describes the modified ABC algorithm combined with self-adaptive searching strategy and artificial immune network operators. In Section 5, the testing of the proposed algorithm through 15 benchmark functions problems is carried out and the simulation results are compared. Finally, conclusions and future works are provided in Section 6.

## 2. Previous Works on the ABC Algorithm

The ABC algorithm imitated the foraging behavior of honeybee and was first applied to numerical optimization problems. However, due to its weaknesses mentioned in Section 1, some researchers proposed many improved strategies. For example, Alatas [19] used different chaotic maps to generate sequences substituting random numbers for different parameters of ABC when producing initial population. Moreover, Gao and Liu [18, 20] also employed both the chaotic systems and opposition-based learning methods to enhance the global convergence. In these two literature works, authors also developed an improved solution search equation which was based on that the bee searched only for the best solution of the previous iteration to improve the exploitation. The experiments derived from a set of 28 benchmark functions demonstrated that the performance of this method was better than the other methods. Unlike these studies mentioned above, inspired by PSO, Zhu and Kwong [21] proposed Gbest-guided ABC algorithm by incorporating the information of the global best solution into the solution search equation to improve the exploitation. Banharnsakun et al. [22] presented a best-so-far method for solution updates in the ABC algorithm, and the searching method based on a dynamic adjustment of search range depending on the iteration was also introduced for scout bees. The test results showed that the proposed method was able to produce higher quality solutions with faster convergence than either the original ABC or the current state-of-the-art ABC-based algorithm.

Besides numerical optimization, the ABC algorithm has been widely used to solve large-scale problems and engineering design optimization. Some representative applications are introduced as follows. Kang et al. [23] used a hybrid ABC algorithm which combines Nelder-Mead simplex method with ABC algorithm for structural inverse analysis problems, and its performance outperforms other heuristic methods. Singh [24] applied the ABC algorithm for the leaf-constrained minimum spanning tree (LCMST) problem and compared the approach against GA, ACO and tabu search. In the literature [24], it was reported that the proposed algorithm was superior to the other methods in terms of solution qualities and computational time. Zhang et al. [25] developed the ABC clustering algorithm to optimally partition *N* objectives into *K* cluster and Deb's rules were used to direct the search direction of each candidate. Pan et al. [26] used the discrete ABC algorithm to solve the lot-streaming flow shop scheduling problem with the criterion of total weighted earliness and tardiness penalties under both the idling and no-idling cases. Samanta and Chakraborty [27] employed ABC algorithm to search out the optimal combinations of different operating parameters for three widely used nontraditional machining (NTM) processes, that is, electrochemical machining, electrochemical discharge machining, and electrochemical micromachining processes. Alejandro et al. [28] used the ABC algorithm in order to find the optimal distribution of material with the aim of establishing a standard time for this duty by examining how this was applied in a local manufacturing plant. The simulation results showed that using this approach might be convenient to set the standard times in the selected company. All these researches illustrated that the ABC algorithm has powerful ability to solve much more complex engineering problems.

## 3. The Original Artificial Bee Colony Algorithm

The artificial bee colony has been inspired by the intelligent behavior of real honey bees. The honey bees in this algorithm are categorized into three groups: employed bees, onlooker bees, and scout bees. The first half of the colony consists of employed bees, and the other half includes the onlookers. Each solution in the search space consists of a set of optimization parameters which represent a food source population. The number of employed bees is equal to the number of food sources around the hive. In other words, for every food source, there is only one employed bee. What is more, onlooker bees wait in the hive and decide on a food source to exploit based on the information shared by the employed bees. Scout bees are translated from a few employed bees whose food source has been exhausted by the bees.

Similar to the other swarm intelligence algorithms, ABC is an iterative process. The units of the original ABC algorithm can be explained as follows.

### 3.1. The Initial Population of Solutions

The initial population of solutions is filled with SN number of randomly generated *D*-dimensional real-valued vectors (i.e., food sources). Each food source is generated as follows:

(1) $$\begin{array}{c} x_{i}^{j}=x_{\min}^{j}+\text{rand}\left(0,1\right)\left(x_{\max}^{j}-x_{\min}^{j}\right), \end{array}$$

where *i* = 1, 2,…, SN, *j* = 1, 2, …, *D*. *x*_min⁡_^*j*^ and *x*_max⁡_^*j*^ are the lower and upper bounds for the dimension*j*, respectively. These food sources are randomly assigned to SN number of employed bees and their fitness is evaluated.

After initialization, the population of the food source is subjected to repeat cycle of the search processes of the employed bees, the onlooker bees, and the scout bees.

### 3.2. The Search Phase of Employed Bees

In this phase, in order to produce a candidate food position from the old one, the ABC uses the following equation:

(2) $$\begin{array}{c} v_{i}^{j}=x_{i}^{j}+\varphi_{i}^{j}\left(x_{i}^{j}-x_{k}^{j}\right), \end{array}$$

where *j* ∈ {1,2,…, *D*} and *k* ∈ {1,2,…, SN} are randomly chosen indexes. Although *k* is determined randomly, it has to be different from *i*. *φ*_*j*_^*i*^ is a random number in the range [−1, 1]. Equation (2) denotes that, within the neighborhood of every food source site represented by *x*_*i*_, a food source *v*_*i*_ is determined by changing one parameter of *x*_*i*_.

Once *v*_*i*_ is obtained, it will be evaluated and compared to *x*_*i*_. A greedy selection is applied between *x*_*i*_ and *v*_*i*_; then the better one is selected depending on fitness values representing the nectar amount of the food sources at *x*_*i*_ and *v*_*i*_. If the fitness of *v*_*i*_ is equal to or better than that of *x*_*i*_, *v*_*i*_ will replace *x*_*i*_ and become a new member of the populations; otherwise *x*_*i*_ is retained.

### 3.3. The Selection Phase of Onlooker Bees

In this phase, each onlooker bee selects one of the food sources depending on the fitness value obtained from the employed bees. The fitness-based probability selection, scheme may be a roulette wheel, ranking based, stochastic universal sampling, tournament selection or another selection scheme. In original ABC, roulette wheel selection scheme is employed described as an equation below:

(3) $$\begin{array}{c} P_{i}=\frac{\text{fit}\left(x_{i}\right)}{\sum_{m=1}^{\text{SN}}\text{fit}\left(x_{m}\right)}, \end{array}$$

where fit(*x*_*i*_) is the fitness value of solution *i*. Obviously, the higher the fit(*x*_*i*_) is, the more probability is that the *i*th food source is selected. After the food source is selected, onlooker bees will go to the selected food source and produce a new candidate position in the neighborhood of the selected food source by using (2).

### 3.4. Scout Bee Phase

In a cycle, after all employed and onlooker bees complete their searches, the ABC algorithm checks if there is any exhausted source to be abandoned. If a position cannot be improved further through a predetermined number of cycles, then that food source is assumed to be abandoned. The scouts can accidentally discover rich, entirely unknown food sources. This operation can be defined as in (4) shown as follows. This process helps avoid suboptimal solutions. The value of predetermined number of cycles is called “*limit*” for abandoning a food source, which is an important control parameter of ABC algorithm:

(4) $$\begin{array}{c} x_{i}=x_{\min}+\text{rand}\left(0,1\right)\left(x_{\max}-x_{\min}\right), \end{array}$$

where *x*_min⁡_ and *x*_max⁡_ are the lower and upper bounds of variable *x*_*i*_.

### 3.5. Main Steps of the Original Artificial Colony Bee Algorithm

Based on the above explanation, there are three control parameters used in the original ABC: the number of the food sources which is equal to the number of employed bees (SN), the value of *limit* and the maximum cycle number (MEN). Detailed pseudocode of the ABC algorithm is given in Algorithm 1 [15].

## 4. Enhancing Artificial Bee Algorithm with Artificial Immune Network

The original version of ABC algorithm is very efficient for multidimensional basic functions. However, the convergence rate of the algorithm is poor when working with some complex multimodal functions and composite functions. Furthermore, due to its poor exploration process, the ABC algorithm easily gets trapped in a local optimum. In order to improve these limitations existing in the ABC algorithm, some modifications inspired by the artificial immune network (ai-Net) algorithm so as to accelerate the convergence rate have been introduced in the search process of the original ABC algorithm. In addition, an improved search mechanism based on the self-adaptive strategy as well as a novel generation method of the initial population is also proposed.

### 4.1. Generation of the Initial Population

One of the modifications in the ABC algorithm is generating effective initial population, which can affect the convergence rate and the quality of the final solution. Generally, random initialization is the most adopted approach to generate initial population, which often makes solutions concentrated in a local area. Chaotic sequences derived from a chaotic map have been proven easy and fast to store; there is no need for storage of long sequences. Recently, chaotic sequences have been used instead of random sequences and shown somewhat good results in many applications. Therefore, chaotic maps are introduced in ABC to improve the global convergence by escaping the local solutions in [19]. Meanwhile, in order to increase the population diversity, similar individuals should be gotten rid of. The main principle is to compare the affinity inspired by ai-Net algorithm between two different individuals. As a result, this work proposes a novel initialization approach which uses chaotic systems and affinity-based compression method to produce initial population. Here, according to the literature [19], sinus map is selected and its equation is defined as follows:

(5) $$\begin{array}{c} cm_{n+1}=2.3{\left(cm_{n}\right)}^{2\sin(\pi cm_{n})},\\ cm_{n}=0,1,2,\ldots ,N, \end{array}$$

where *n* is the iteration counter and *N* is the maximum number of chaotic iterations. Furthermore, the affinity equation between two different individuals is defined as Euclidean distance shown in (6):

(6) affinity(xi,xk)=∑j=1D(xij−xkj)2,(i≠k, j∈(1,2,…D))>ξ,

where *ξ* is a threshold value defined in advance so as to control the difference between two individuals. *D* is the number of optimization parameters. Based on these operators, we propose the following algorithm to generate initial population and its corresponding pseudocode is given in Algorithm 2.

### 4.2. An Improved Search Mechanism Based on the Self-Adaptive Strategy

As mentioned above, the original ABC algorithm is good at exploration but poor at exploitation due to two reasons. On the one hand, in (2), the coefficient *φ*_*j*_^*i*^ is a uniform random number in [−1,1] and *x*_*j*_^*k*^ is a random individual in the population; therefore, the search process of solutions illustrated by (2) is random enough for exploration [21]. On the other hand, a greedy selection mechanism is employed between the old and candidate solutions, which may easily make solutions get trapped in a local optimal. What is more, the slower convergence rate of the algorithm is another limitation when working with some complex composite functions. As a result, we introduce a self-adaptive strategy to improve its search process and the detailed explanations are as follows.

Firstly, we can see from (2) that there is only one different element between the candidate solution and the old one (i.e., the *j*th element). This search strategy may be efficient in earlier iterations. However, when the solution approaches to a local optimum, its search efficiency becomes poor in later iterations. To handle this limitation, similar to [29, 30], we introduce a parameter *L* to control the difference between the candidate solution and the old one, where how to choose the value of the parameter *L* is very important. Generally, the higher the value of *L* is, the more information is brought into the candidate solution. In the literature [18], the parameter *L* is a fixed constant and chosen according to simulation experiments. Different from this approach mentioned above, this paper proposes a self-adaptive adjustable strategy to determine the parameter *L* and the corresponding search equation is given below:

(7) $$\begin{array}{c} v_{i}^{l}=x_{i}^{l}+cm_{i}^{l}\left(x_{i}^{l}-x_{k}^{l}\right),\;l=\left\{1,2,\ldots ,L\right\}, \end{array}$$

(8) $$\begin{array}{c} L=1+||D\left(\frac{\text{iteration}}{2\times \text{MEN}}\right)||, \end{array}$$

where *cm*_*i*_^*l*^ is a chaotic map defined by (5). *D* is the number of optimization parameters. A symbol ||·|| denotes a rounding operator. We can see from (8) that onlooker bees will search better solutions in only one direction in the first iteration and they will search in the whole space with the increase in the value of *L* when the solutions are closely to the local optimum in later iterations. However, we limit the value of *L* no more than ||1 + *D*/2||. This is because, with the higher value of *L*, the solution generated by the search equations (7)-(8) is more likely random search operator. As a result, (7)-(8) will dynamically adjust the position of onlooker bees by allowing them to explore with a wider search space in later iterations. As the number of the iterations increases, the corresponding search space of onlooker bees will also increase.

The second modification in the ABC algorithm lies in the selection probability of onlooker bees associated with the food source. In the original ABC algorithm, the fitness values obtained from the employed bees are adopted to determine the selection probabilities of onlooker bees. Nevertheless, the fitness comparisons among different individuals only reflect the qualitative information. In this work, based on the idea of the fitness evolution, we introduce an environment factor *η*_*i*_ corresponding to every food source so as to evaluate its exploitation potential. In other words, the parameter *η*_*i*_ is used to evaluate quantitatively the environment situations of exploitation for every food source. As the number of iterations increases, the higher the value of *η*_*i*_, the better exploitation environment it corresponds to. At this moment, more onlooker bees will follow the corresponding employed one to its food source position with higher nectar amount in order to accelerate exploitation efficiency. Conversely, if the value of *η*_*i*_ is lower, its corresponding solution lies in the worse exploitation environment, which means that it is difficult to find out a better solution around the old food source and less onlooker bees will be attracted by the employed one. As a result, how to define *η*_*i*_ needs explain. Generally, the parameter *η*_*i*_ is associated with both a fitness change amount Δ*f* and a count accumulator *C*, where Δ*f* denotes the fitness difference associated with the same food source between two adjacent generations which reflects the exploitation potential of the corresponding food source position. Besides, a count accumulator *C* will be explained in the following text. The equations below reflect the fitness change amount Δ*f* between two adjacent generations:

(9) $$\begin{array}{c} \Delta f_{t}\left(x_{i}\right)=\left|\frac{\text{fi}{\text{t}}_{t}\left(x_{i}\right)-\text{fi}{\text{t}}_{t-1}\left(x_{i}\right)}{\text{fi}{\text{t}}_{t-1}\left(x_{i}\right)}\right| \end{array}$$

(10) $$\begin{array}{c} \Delta f_{t}^{\prime }\left(x_{i}\right)=\exp\left[\Delta f_{t}\left(x_{i}\right)\right]=\log\left|\frac{\text{fi}{\text{t}}_{t}\left(x_{i}\right)-\text{fi}{\text{t}}_{t-1}\left(x_{i}\right)}{\text{fi}{\text{t}}_{t-1}\left(x_{i}\right)}\right|, \end{array}$$

where the parameter *t* denotes the number of iterations and the symbol | | means absolute value sign. As can be seen from (9), the value range of Δ*f* is between 0 and 1. When the value of Δ*f* is higher, it means the corresponding food source has a higher exploitation potential and is largerly possible to find out a better solution and vice versa. However, at later iterations, the value of Δ*f* will be very small and need appropriate amplification. As a result, we adopt power function to amplify Δ*f* here and take the number *e* as its base. Due to this reason, (9) is substituted for (10).

In addition, if Δ*f* is less than a given small value in advance (i.e., Δ*f*≤Δ*f*_0_, and Δ*f*_0_ is the threshold of Δ*f*), the improved ABC algorithm may trigger a count accumulator called *Counter* (represented by *C* in this paper) which is used to record how many times the quality of the solution has not improved (it corresponds to the number of iterations in the algorithm). The following equation is used to express the count accumulator *C* at the *t*th iteration. Generally, the larger the value of *C*, the less exploitation potential that the food source position corresponds to:

(11) $$\begin{array}{c} C\left(t\right)=\{\begin{array}{cc} C\left(t-1\right)+T\left(t\right)=\sum_{i=s}^{t}T\left(i\right), & T\left(t\right)=1,\\ 0, & T\left(t\right)=0, \end{array} \end{array}$$

where *T* represents a pulse signal. When Δ*f* > 0 or Δ*f* ≥ Δ*f*_0_, *T* = 0; conversely, when Δ*f* = 0 or Δ*f* ≤ Δ*f*_0_, *T* = 1. It means that

(12) $$\begin{array}{c} T\left(t\right)=\{\begin{array}{cc} 1, & \left(\Delta f\left(t\right)=0\right)\;\;\text{or}\;\;\left(\Delta f\left(t\right)\leq \Delta f{\left(t\right)}_{0}\right),\\ 0, & \left(\Delta f\left(t\right)>0\right)\;\;\text{or}\;\;\left(\Delta f\left(t\right)\geq \Delta f{\left(t\right)}_{0}\right). \end{array} \end{array}$$

Note that, according to the performance requirement of specific problems, the maximum times of the stagnation of global extrema, *C*_max⁡_, should be defined in advance, which means, if a minimum of a function has not been updated for continuous *C*_max⁡_ iterations, the current exploitation area has few potentials to find out the better solution and computation resources should be redistributed. Generally, we define that the *C*_max⁡_ is equal to or not less than 5. In addition, *C*(*t*) is normalized for simplifying the problem and *C*′(*t*) can be obtained which is expressed as follows:

(13) $$\begin{array}{c} C^{\prime }\left(t\right)=\frac{\sum_{i=s}^{t}T\left(i\right)+1}{C_{\max}+1}. \end{array}$$

On the basis of the definitions of the fitness change amount Δ*f*and the count accumulator *C*, the environment factor *η* is defined as follows:

(14) $$\begin{array}{c} \eta \left(t\right)=\frac{\Delta f^{\prime }\left(t\right)}{C^{\prime }\left(t\right)}. \end{array}$$

Submitting (10) and (13) into (14), we can achieve the following equation:

(15) $$\begin{array}{c} \eta \left(t\right)=\frac{\left(C_{\max}+1\right)\cdot \exp\left|\left(\text{fi}{\text{t}}_{t}\left(x_{i}\right)-\text{fi}{\text{t}}_{t-1}\left(x_{i}\right)\right)/\text{fi}{\text{t}}_{t-1}\left(x_{i}\right)\right|}{\sum_{i=s}^{t}T\left(i\right)+1}. \end{array}$$

In accordance with the expression of the environment factor *η*(*x*_*i*_) corresponding to every food source, the selection probability of onlooker bees associated with the food source can be substituted with the following equation:

(16) $$\begin{array}{c} P_{i}=\frac{\eta_{t}\left(x_{i}\right)}{\sum_{m=1}^{\text{SN}}\eta_{t}\left(x_{m}\right)}. \end{array}$$

### 4.3. Enhancing Convergence Efficiency with Artificial Immune Network Operators

In the basic ABC system, artificial bees fly around in the search space. Some (like employed and onlooker bees) choose food source depending on the experience of themselves and their nest mates and then adjust their positions, but others (like scouts) fly and choose the food sources randomly without using experience. If the nectar amount of a new source is higher than that of the previous one in their memory, they memorize the new food source position and forget the previous one. Thus, the ABC system combines local search methods, carried out by employed and onlooker bees, with global search methods, managed by Karaboga and Basturk [15]. However, unlike the ABC system, the concept of artificial immune system (AIS) was originated by observing how the defense mechanism of natural immune system protects against attacks by antigens. There are numerous AIS algorithms developed for a variety of applications, where artificial immune network (aiNet for short) is a typical one and its algorithms and models are originally proposed to perform information compression and data clustering based on artificial immune system (AIS) theory [31]. Immune network-based algorithms are similar to clonal selection algorithms in that they both measure the goodness of antibodies by affinities, and both methods include a series of steps for selecting, cloning, and mutating antibodies. The major difference is that the immune network-based algorithms are represented by network graph structures [32]. Compared with other ones, the immune network-based algorithms employ extra procedures of antibody pruning and suppressing. This allows the models to generate a smaller, less-redundant population of antibody representatives, which is desirable for solving multimodal function optimization. Comparing ABC optimization with ai-Net algorithm, we can see that the advantages of ABC optimization lie in its neighborhood search method according to the profitability of food sources. However, ai-Net algorithm adopts fixed clonal individuals to perform local search which has certain blindness. In addition, due to introducing network compression, negative selection, and other operators, ai-Net can maintain the diversity of the population and reduce the possibility of being trapped into a local minimum. Unlike the ai-Net algorithm, ABC optimization maintains population diversity through random search of scout bees, which has obvious limitation. Based on the analysis mentioned above, if network compression and negative selection deriving from ai-Net algorithm are introduced into ABC optimization, this improved one may have a powerful and efficient multimodal searching ability as well as good stabilization. The detailed process is as follows.

Different employed bee individual corresponds to different food source position. In order to eliminate redundant and similar food sources, negative selection and network compression are used to compare with the similarities among various individuals. The Euclidean distance of two employed bee individuals *X*_*i*_ and *X*_*k*_ is adopted as shown in (17):

(17) $$\begin{array}{c} d\left(X_{i},X_{k}\right)=\sqrt{\sum_{j=1}^{D}{\left(X_{i}^{j}-X_{k}^{j}\right)}^{2}}\;\left(i\neq k\right). \end{array}$$

In order to simplify the problem, the affinity concept is introduced which is obtained by the following equation using the normalization method:

(18) $$\begin{array}{c} A\left(X_{i},X_{k}\right)=\frac{1}{1+d\left(X_{i},X_{k}\right)}, \end{array}$$

where the value range of *A*(*X*_*i*_ *X*_*k*_) is between 0 and 1. The smaller the value of *A*(*X*_*i*_ *X*_*k*_) is, the larger the value of *A*(*X*_*i*_ *X*_*k*_) is, which means that two different employed bee individuals have a higher similarity. Specially, when *A*(*X*_*i*_ *X*_*k*_) equals 1, these two ones are identical. According to negative selection and network compression operators, redundant and similar food sources should be eliminated. We predefine a threshold value, *ε*, so as to realize wipe-off of redundant individuals. It also means when *A*(*X*_*i*_ *X*_*k*_) is equal to or great than *ε*, we think these two ones are identical and only one can be retained and other should be wiped off. Repeat this process until the affinity of any two individuals in a population is less than *ε*. In doing so, the population size may be reduced. Nevertheless, in order to keep the population size unchanged, the same number of new individuals need generating randomly. Through negative selection and network compression operators, the exploitation efficiency will be improved and the corresponding convergence rate of the algorithm will also be accelerated.

### 4.4. Main Steps of the Enhanced Artificial Bee Colony Algorithm

Based on the above analysis, three main improvements including novel generation of initial population, self-adaptive searching strategy, and redundant individual compression operator are presented and the detailed pseudo-code is given in Algorithm 3.

## 5. Experimental Studies on Function Optimization Problems

### 5.1. Benchmark Functions and Parameter Settings

In this section, numerical experiment is used to test the performance of the enhanced ABC (shorthand for EABC) proposed in this paper. Summarized in Table 1 are the 15 scalable benchmark functions. *f*_1_~*f*_10_ are continuous unimodal functions. *f*_11_~*f*_15_ are multimodal functions and the number of their local minima increases exponentially with the problem dimension.

In order to testify the performance of different intelligent algorithms, we compare the EABC with the standard ACO, PSO, and ABC. In all simulations, the population size of ACO, PSO, ABC, and EABC is 50. The maximum number of function evaluations (FE) is set to 5000. The threshold value of the affinity, *ε*, is 0.9. Other related parameter values of ACO, PSO, and ABC are referred in the literature [17]. All experiment results reported are obtained based on 30 independent runs. The experiment results are the best, worst, mean, and standard deviation of the statistical experimental data.

### 5.2. Simulation Results

The performance on the solution accuracy of EABC is compared with that of ACO, PSO, and ABC.  Table 2 shows the optimization of the 15 benchmark functions obtained in the 30 independent runs by each algorithm and some interesting results can be found in Table 2.

Firstly, almost all algorithms have identical performance on most of unimodal functions *f*_1_ to *f*_4_, *f*_7_, *f*_8_, and *f*_14_. However, on other functions, these four algorithms show different performance, especially for multimodal ones such as *f*_11_, *f*_12_, *f*_13_, and *f*_15_. Fox example, on function *f*_15_, the best values obtained by ACO, PSO ABC, and EABC are −10296, −6993.47, −12566.9, and −152568.7, respectively. It means that EABC can be efficiently applied for solving multimodal and multidimensional function optimization problems due to its abundant operators such as clonal selection and negative selection which outperforms ACO, PSO, and ABC. In addition, on the one hand, on basic unimodal functions, both the basic ABC and EABC have almost identical solving performance; on the other hand, on the multimodal functions, these two ones display huge difference.

Secondly, the EABC algorithm can find optimal or closer-to-optimal solutions on the complex multimodal functions *f*_11_, *f*_12_, *f*_13_, and *f*_14_. Although the result of multi-dimension function *f*_15_ is slightly far from the known global optimum, the EABC is superior to the other algorithms all the same. At the same time, for almost all benchmark functions, standard deviations of the EABC obtained from the statistical experimental data are no greater than those of others expect for *f*_10_. In addition, the differences of EABC between the best and worst solutions for these 15 benchmark functions are relatively smaller than those of others in the 30 independent simulation runs. All these mean that the EABC algorithm has better robustness than others. It is also clear that EABC can work better in almost all cases and gets better performance than ACO, PSO, and ABC.

Summarizing the statements mentioned above, the EABC can prevent bees from being trapped into the local minimum, accelerate convergence process, search with more efficiency, and improve exploitation abilities for basic ABC.

### 5.3. Analysis and Discussion

**Figure 1:**
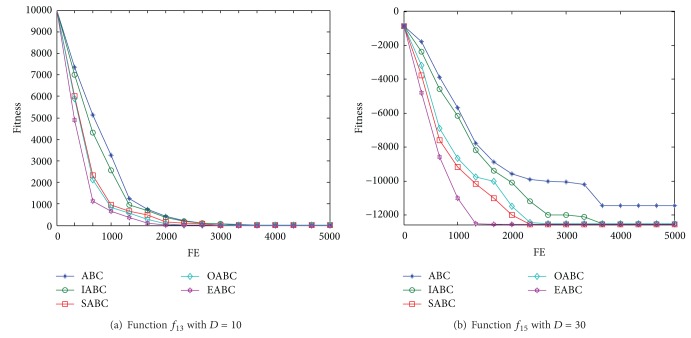
Convergence speed of the different ABCs on the two test functions (*f*_13_, *f*_15_).

In this section, the effects of each modification on the performance of EABC are discussed. First of all, corresponding to three modifications, we named the basic ABC with the proposed initialization as IABC, the one with the proposed self-adaptive searching strategy as SABC, and the one with the proposed immune operators as OABC. We compare the convergence speed of these different ABCs through two complex high-dimension multimodal functions *f*_13_ and *f*_15_ in order to find the contributions of three modifications in EABC to improve the performance of the algorithm, respectively. The corresponding simulation results are shown in Figure 1. We can see from Figure 1 that IABC, SABC and OABC outperform the basic ABC, which means that the three modification measures mentioned in Section 4 have positive effect on the convergence speed of the algorithm. In addition, SABC, and OABC are obviously superior to IABC, which implies that searching strategy and immune operators play more important roles than that of initialization. However, it is difficult to compare the contributions between searching strategy and immune operators on the two test functions; the reasons may be that the characteristic of test functions will also affect the problem-solving efficiency of the algorithm.

## 6. Conclusion

In this paper, we have proposed an enhanced artificial bee colony algorithm, called EABC through introducing self-adaptive searching strategy and artificial immune network operators. Subsequently, a suite of unimodal or multimodal benchmark functions are used to testify the performance of the proposed algorithm. The simulation results illustrate that the EABC algorithm outperforms ACO, PSO, and the basic ABC.

The future work includes the studies on how to apply EABC to more complex discrete dynamic optimization problems including product design optimization problem, dynamic project scheduling problem, and data clustering problem.

## Figures and Tables

**Algorithm 1:**
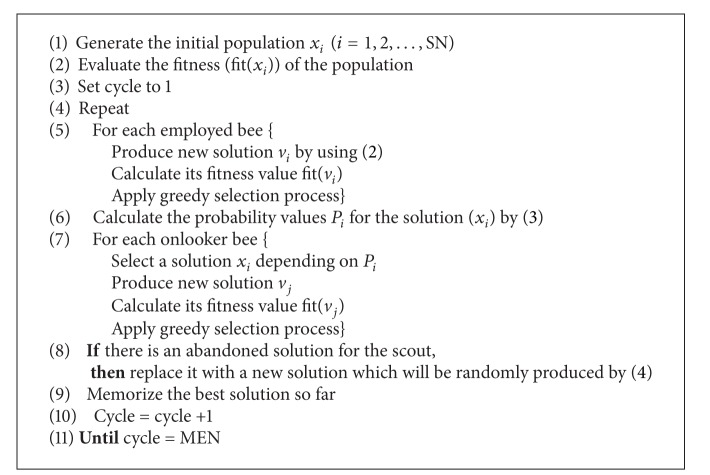
Pseudocode of main body of ABC algorithm.

**Algorithm 2:**
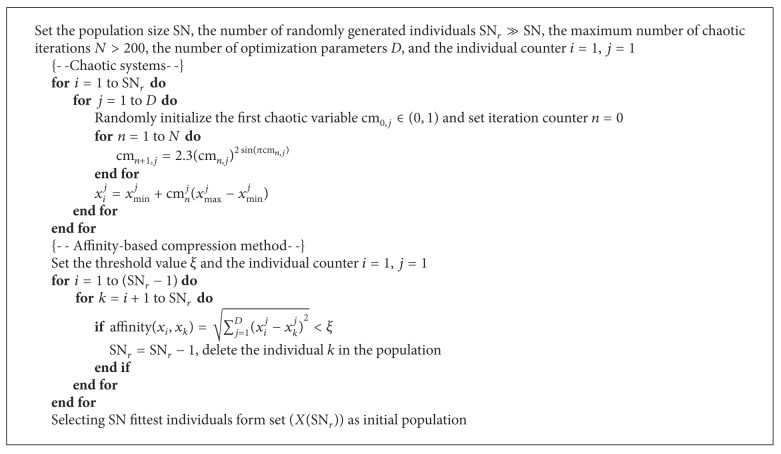
Modified initialization step of ABC algorithm.

**Algorithm 3:**
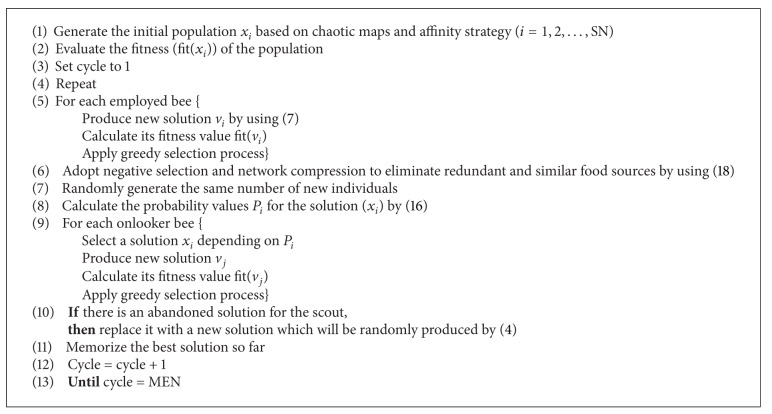
Pseudocode of main body of the enhanced ABC algorithm.

**Table 1:** Benchmark functions used in experiments.

Number	Function	Dimension	Property	Range	Min
1	$f_{1}(x)=\overset{D}{\underset{i=1}{\sum }}x_{i}^{2}$	30	Unimodal	[−100, 100]	0
2	$f_{2}(x)=\overset{D}{\underset{i=1}{\sum }}ix_{i}^{2}$	30	Unimodal	[−10, 10]	0
3	$f_{3}(x)=25+\overset{D}{\underset{i=1}{\sum }}\left\lfloor x_{i}\right\rfloor$	5	Unimodal	[−5.12, 5.12]	0
4	$f_{4}(x)=\overset{D}{\underset{i=1}{\sum }}{\left(\left\lfloor x_{i}+0.5\right\rfloor \right)}^{2}$	30	Unimodal	[−100, 100]	0
5	$f_{5}(x)=\overset{D}{\underset{i=1}{\sum }}ix_{i}^{4}+\text{random}(0,1)$	30	Unimodal	[−1.28, 1.28]	0
6	*f*_6_(*x*) = max⁡_*i*_{||*x*_*i*_||, 1 ≤ *i* ≤ *D*}	30	Unimodal	[−100, 100]	0
7	$f_{7}(x)=-\prod_{i=1}^{D}\cos\left(x_{i}\right)\exp\left(-\overset{D}{\underset{i=1}{\sum }}{\left(x_{i}-\pi \right)}^{2}\right)$	2	Unimodal	[−100, 100]	−1
8	$f_{8}(x)=0.26\overset{D}{\underset{i=1}{\sum }}x_{i}^{2}-0.48\prod_{i=1}^{D}x_{i}$	2	Unimodal	[−10, 10]	0
9	$f_{9}(x)={\left(x_{1}-1\right)}^{2}+\overset{D}{\underset{i=2}{\sum }}i{\left(2x_{i}^{2}-x_{i-1}\right)}^{2}$	30	Unimodal	[−10, 10]	0
10	$f_{10}\left(x\right)=\overset{D}{\underset{i=1}{\sum }}100{\left(x_{i+1}-x_{i}^{2}\right)}^{2}+{\left(1-x_{i}\right)}^{2}$	30	Unimodal	[−30, 30]	0
11	$f_{11}(x)=\overset{D}{\underset{i=1}{\sum }}\left(x_{i}-10\cos\left(2\pi x_{i}\right)+10\right)$	30	Multimodal	[−5.12, 5.12]	0
12	$f_{12}(x)=\frac{1}{4000}\left(\overset{D}{\underset{i=1}{\sum }}{\left(x_{i}-100\right)}^{2}\right)-\left(\prod_{i=1}^{D}\cos\left(\frac{x_{i}-100}{\sqrt{i}}\right)\right)+1$	30	Multimodal	[−600, 600]	0
13	$f_{13}\left(x\right)=10\times D+\overset{D}{\underset{i=1}{\sum }}\left(x_{i}^{2}-10\cos\left(2\pi x_{i}\right)\right)$	10	Multimodal	[−50, 50]	0
14	$f_{14}(x)=0.5+\frac{\sin^{2}\left(\sqrt{\sum_{i=1}^{D}x_{i}^{2}}\right)-0.5}{{\left(1+0.001\left(\sum_{i=1}^{D}x_{i}^{2}\right)\right)}^{2}}$	2	Multimodal	[−100, 100]	0
15	$f_{15}\left(x\right)=\overset{D}{\underset{i=1}{\sum }}-x_{i}\sin\left(\sqrt{||x_{i}||}\right)$	30	Multimodal	[−500, 500]	−12569.5

**Table 2:** Benchmark functions used in experiments for testing the performances of EABC, ACO, PSO, and ABC.

Function number	Min		ACO	PSO	ABC	EABC
$f_{1}(x)=\overset{D}{\underset{i=1}{\sum }}x_{i}^{2}$	0	Best	0	0	0	0
		Worst	0	0	0	0
		Mean	0	0	0	0
		SD	0	0	0	0

$f_{2}(x)=\overset{D}{\underset{i=1}{\sum }}ix_{i}^{2}$	0	Best	0	0	0	0
		Worst	0	0	0	0
		Mean	0	0	0	0
		SD	0	0	0	0

$f_{3}(x)=25+\overset{D}{\underset{i=1}{\sum }}\left\lfloor x_{i}\right\rfloor$	0	Best	0	0	0	0
		Worst	0	0	0	0
		Mean	0	0	0	0
		SD	0	0	0	0

$f_{4}(x)=\overset{D}{\underset{i=1}{\sum }}{\left(\left\lfloor x_{i}+0.5\right\rfloor \right)}^{2}$	0	Best	0	0	0	0
		Worst	0	0	0	0
		Mean	0	0	0	0
		SD	0	0	0	0

$f_{5}(x)=\overset{D}{\underset{i=1}{\sum }}ix_{i}^{4}+\text{random}(0,1)$	0	Best	0	0	0	0
		Worst	0.00289	0.00321	0.00543	0.00422
		Mean	0.00136	0.00116	0.00300	0.00196
		SD	0.00219	0.00276	0.00387	0.00208

*f*_6_(*x*) = max⁡_*i*_{||*x*_*i*_||, 1 ≤ *i* ≤ *D*}	0	Best	0	0	0	0
		Worst	0.00246	0.00305	0.00110	0.00400
		Mean	0.00180	0.00156	0.0066	0.00210
		SD	0.00039	0.00058	0.00092	0.00037

$f_{7}(x)=-\prod_{i=1}^{D}\cos\left(x_{i}\right)\exp\left(-\overset{D}{\underset{i=1}{\sum }}{\left(x_{i}-\pi \right)}^{2}\right)$	−1	Best	−1	−1	−1	−1
		Worst	−1	−1	−1	−1
		Mean	−1	−1	−1	−1
		SD	0	0	0	0

$f_{8}(x)=0.26\overset{D}{\underset{i=1}{\sum }}x_{i}^{2}-0.48\prod_{i=1}^{D}x_{i}$	0	Best	0	0	0	0
		Worst	0	0	0	0
		Mean	0	0	0	0
		SD	0	0	0	0

$f_{9}(x)={\left(x_{1}-1\right)}^{2}+\overset{D}{\underset{i=2}{\sum }}i{\left(2x_{i}^{2}-x_{i-1}\right)}^{2}$	0	Best	0.66667	0.6667	0	0
		Worst	0.66667	0.6667	0	0
		Mean	0.66667	0.6667	0	0
		SD	0.00001	0.00001	0	0

$f_{10}(x)=\overset{D}{\underset{i=1}{\sum }}100{\left(x_{i+1}-x_{i}^{2}\right)}^{2}+{\left(1-x_{i}\right)}^{2}$	0	Best	8.7513	10.5433	19.6788	9.1578
		Worst	32.4215	24.6711	54.2333	26.9874
		Mean	18.2039	15.0886	33.1227	17.3558
		SD	5.0361	24.1702	154.1443	11.4774

$f_{11}(x)=\overset{D}{\underset{i=1}{\sum }}\left(x_{i}-10\cos\left(2\pi x_{i}\right)+10\right)$	0	Best	52.6677	43.5774	0	0
		Worst	53.2331	44.1131	0	0
		Mean	52.9226	43.9771	0	0
		SD	4.5649	11.7286	0	0

$f_{12}(x)=\frac{1}{4000}\left(\overset{D}{\underset{i=1}{\sum }}{\left(x_{i}-100\right)}^{2}\right)-\left(\prod_{i=1}^{D}\cos\left(\frac{x_{i}-100}{\sqrt{i}}\right)\right)+1$	0	Best	0.01470	0.017112	0.008531	0
		Worst	0.01499	0.017989	0.017356	0
		Mean	0.01479	0.017391	0.011447	0
		SD	0.00296	0.020808	0.001223	0

$f_{13}(x)=10\times D+\overset{D}{\underset{i=1}{\sum }}\left(x_{i}^{2}-10\cos\left(2\pi x_{i}\right)\right)$	0	Best	4.56781	21.44755	3.34788	0
		Worst	8.77993	39.55741	10.91447	0
		Mean	5.85411	26.3991	5.59331	0
		SD	13.1142	155.6380	10.04216	0

$f_{14}(x)=0.5+\frac{\sin^{2}\left(\sqrt{\sum_{i=1}^{D}x_{i}^{2}}\right)-0.5}{{\left(1+0.001\left(\sum_{i=1}^{D}x_{i}^{2}\right)\right)}^{2}}$	0	Best	0	0	0	0
		Worst	0	0	0	0
		Mean	0	0	0	0
		SD	0	0	0	0

$f_{15}(x)=\overset{D}{\underset{i=1}{\sum }}-x_{i}\sin\left(\sqrt{||x_{i}||}\right)$	−12569.5	Best	−10296	−6993.47	−11566.9	−12568.7
		Worst	−10237	−6883.33	−11498.8	−12514.3
		Mean	−10266	−6909.12	−11544.1	−12551.1
		SD	521.849	457.9577	125.4471	101.3217
